# Longitudinal Analysis of Quadriceps Muscle Strength in Patients with Previous COVID-19 Hospitalization and in Patients with Post-Acute Sequelae following Mild COVID-19

**DOI:** 10.3390/nu14204319

**Published:** 2022-10-15

**Authors:** Anouk A. F. Stoffels, Esther L. van Voorthuizen, Hieronymus W. H. van Hees, Jeannette B. Peters, Hanneke A. C. van Helvoort, Nicol C. Voermans, Jonne Doorduin, Bram van den Borst

**Affiliations:** 1 Department of Pulmonary Diseases, Radboud University Medical Center, 6500 HB Nijmegen, The Netherlands; 2 Department of Neurology, Donders Institute for Brain, Cognition and Behaviour, Radboud University Medical Center, 6500 HB Nijmegen, The Netherlands

**Keywords:** COVID-19, post-COVID syndrome, muscle weakness, myopathy, rehabilitation

## Abstract

Muscle weakness is a prominent symptom in post-acute sequelae of COVID-19 (PASC). However, few studies have objectively and longitudinally assessed muscle strength after varying COVID-19 severity grades. This observational study aimed to explore the prevalence, determinants, and 1.5 years change of quadriceps muscle weakness in 98 patients discharged from COVID-19 hospitalization and in 50 patients with PASC following mild COVID-19. Isometric quadriceps maximal voluntary contraction (MVC) was assessed on a computerized dynamometer at three visits. Also, in a subgroup of 14 post-COVID-19 patients with quadriceps muscle weakness, muscle thickness and echo intensity were determined by muscle ultrasound of nine upper and lower extremity muscles. Muscle weakness was found in 59% of post-hospitalized patients and in 65% of those with PASC following mild COVID-19 at ~14 weeks after acute COVID-19. Whereas during ~1.5 years follow-up MVC modestly improved, muscle weakness prevalence remained unchanged. Hospital length of stay and diabetes mellitus were identified as possible predictors of muscle weakness following COVID-19 hospitalization. No predictors could be identified in those with PASC following mild COVID-19. Ultrasound outcomes revealed no large structural abnormalities. In conclusion, clinically relevant muscle weakness is common after COVID-19 and its long-term improvement is poor. Future studies with relevant control groups are warranted to confirm our data.

## 1. Introduction

Worldwide, by October 2022, over 619 million cases of coronavirus disease-19 (COVID-19), caused by Severe Acute Respiratory Syndrome coronavirus-2 (SARS-CoV-2), have been registered [1]. Symptoms and severity of acute COVID-19 vary widely, ranging from asymptomatic or mild to critical illness [2]. Although the vast majority recover quickly [3], it has been globally observed that a substantial proportion experiences long-lasting symptoms [4], also termed ‘long COVID’ or post-acute sequelae of COVID-19 (PASC).

PASC comprises a constellation of symptoms in which fatigue and muscle weakness are among the most prominent [5,6]. In patients after discharge from COVID-19 hospitalization, research has shown that quadriceps muscle weakness was present in 60% at 3 months [7], and fatigue and muscle weakness were reported by 63% at 6 months [8]. In general, hospitalization itself, in conjunction with (forced) physical inactivity, inflammation induced by the illness, loss of muscle mass, and malnutrition, has been associated with a reduction of knee extensor strength [9]. More specifically, in patients requiring treatment in the intensive care unit (ICU), critical illness polyneuromyopathy is a well-known cause of muscle weakness [10], and its various subtypes may affect almost half of all ICU patients regardless of the primary cause of critical illness [11]. Interestingly, an increased risk of self-reported reduced strength in arms/legs has recently also been reported among predominantly non-hospitalized post-COVID-19 patients [12]. Seemingly, peripheral muscle weakness may be a common phenomenon following COVID-19 regardless of whether hospitalization was required in the acute phase.

Studies evaluating the prevalence, risk factors, and long-term course of muscle weakness after varying degrees of COVID-19 severity are still scarce. Insights into these elements are projected to aid in our aetiological understanding of muscle weakness following COVID-19 and might provide guidance for optimizing rehabilitation strategies. Therefore, the present study aimed to explore the prevalence, determinants, and 1.5 years change of objectively assessed quadriceps muscle weakness in two post-COVID-19 cohorts: (1) patients after discharge from COVID-19 hospitalization, and (2) patients with PASC following mild COVID-19 without hospitalization. The secondary aim was to evaluate whether muscle ultrasound thickness and echo intensity abnormalities were present in a subgroup of post-COVID-19 patients with quadriceps muscle weakness. We hypothesized (1) to find high prevalence of muscle weakness in both cohorts, (2) that severity markers of acute infection predict muscle weakness, (3) that muscle strength would improve over time, and (4) that skeletal muscle ultrasound would detect low muscle thickness and high echo intensity in those with quadriceps muscle weakness.

## 2. Materials and Methods

This observational study was conducted at the outpatient COVID-19 aftercare facility at Radboud university medical centre, Nijmegen, The Netherlands, between June 2020 and December 2021. Patients had either been discharged from hospitalization for moderate-to-critical COVID-19 (post-hospitalized cohort) or were referred by their general practitioner for the analysis of PASC following mild COVID-19 for which no hospitalization was required (PASC after mild COVID-19 cohort). Both cohorts were offered three visits over a period of ~1.5 years, and will be addressed separately throughout this manuscript. Inclusion criteria were: age ≥18 years old and proof of previous COVID-19 (based on positive SARS-CoV-2 real-time polymerase chain reaction or serology).

### 2.1. Quadriceps Muscle Strength Assessment

At all visits, isometric quadriceps muscle strength of the right leg was determined (unless not possible due to injury) using a computerized dynamometer (Biodex System 4 Pro, Biodex Medical Systems, Inc., Shirley, NY, USA). Patients were positioned as previously described [13]. The arms were crossed over the chest and straps were used over the pelvis and thigh. After a warm-up protocol of five sub-maximal contractions, patients performed three maximal unilateral isometric knee extensions for five seconds at a knee angle of 60°, interspersed with 15 seconds of rest. To avoid submaximal performance or outliers, sets of measurements with a coefficient of variation >10% between the three knee extensions were determined invalid and were excluded from analyses [14]. Maximal voluntary contraction (MVC) was defined as the highest peak torque and was expressed in Newton meter (Nm) and as a percentage of the predicted value according to age, height, gender, and body mass derived from a group of healthy individuals [14]. Quadriceps muscle weakness was defined as MVC below the lower limit of normal.

### 2.2. Acute COVID-19 Severity Markers

Hospital length of stay, ICU admission, chest computed tomography pulmonary COVID-19 severity score at admission [15], peak inflammatory markers during hospitalization (highest C-reactive protein [CRP], d-dimer and ferritin which were routinely assessed every other day), and COVID-19 medication were collected from patients’ medical records.

### 2.3. Data Collection and Measurements at Visit 1

At visit 1, lung function, body mass index (BMI), body composition, nutritional status, dyspnea, physical functioning, fatigue, physical capacity, and serum CRP levels were assessed. Spirometry and single-breath lung diffusion capacity (MasterSCreen PFT; Jaeger, Würzburg, Germany) were performed and outcomes were expressed according to reference values [16,17,18]. Bio-electrical impedance analysis (Bodystat 500; EuroMedix, Leuven, Belgium) was applied, excluding patients with peripheral oedema or pacemaker. Fat-free mass index (FFMi) was calculated as fat-free mass divided by height squared, and reference values were applied to determine low FFMi [19]. Nutritional status, dyspnea, physical functioning, and fatigue were assessed using the Patient-Generated Subjective Global Assessment Short Form (PG-SGA SF) [20], the modified Medical Research Council (mMRC) dyspnea scale [21], the physical functioning domain of the 36-item Short Form Health Survey (SF-36) [22], and the Checklist Individual Strength (CIS-fatigue) [23], respectively. Physical capacity was assessed by the 6-min walking test [24], and CRP levels were determined in the central hospital laboratory. Finally, age, gender, smoking status, comorbidities, and date of first COVID-19 symptoms were collected from patients’ medical records.

### 2.4. Muscle Ultrasound

Muscle ultrasound was performed in a selection of patients with quadriceps muscle weakness who had no relevant comorbidities and a BMI below 30 kg/m^2^. The latter criterion was applied because of the range of normative values for muscle thickness and echo intensity available in our centre. Ultrasound measurements were executed within three months after quadriceps muscle strength assessment by experienced technicians using an Esaote MyLabTwice ultrasound machine (Esaote SpA, Genoa, Italy) with a 3–13 MHz broadband linear transducer (LA533) according to current standards [25]. M. rectus femoris, m. vastus lateralis, m. tibialis anterior, medial head of m. gastrocnemius, m. peroneus tertius, m. deltoideus, m. biceps brachii, m. flexor carpi radialis and m. interosseus dorsalis I were examined unilaterally. Each muscle was measured three times using a standardized clinical protocol based on anatomical landmarks. A custom-developed software package (Matlab 2013b, Mathworks, Natick, MA, USA) was used to analyse muscle ultrasound images. First, a region of interest was manually drawn in the cross-sectional area of the muscle. Second, absolute echo intensity was calculated as the mean grey level of the pixels within the region of interest. Third, an average was determined from the three images for each muscle. Muscle thickness was calculated by measuring the distance between the upper and lower boundary of the muscle (often the overlying fascia or underlying bone). Z-scores of echo intensity and muscle thickness were calculated by the ratio of the difference between the measured value and the predicted value with the residual standard deviation. A z-score ≥ 2 for echo intensity (indicating inflammation, fibrosis, or fatty infiltration) and a z-score ≤ −2 for muscle thickness (indicating atrophy) were defined as abnormal. The predicted value per muscle for echo intensity and muscle thickness was calculated using a multiple linear regression equation including age, sex, weight, height, and BMI [26], which was developed in our centre from muscle ultrasound images from 80 healthy subjects with an even distribution of age (range 0–90 years) and gender.

### 2.5. Statistical Analysis

Continuous variables were presented as mean ± standard deviation or median (interquartile range) as appropriate. Categorical data were presented as numbers and percentages. Differences in subgroups were assessed using an independent-samples *t*-test, Mann–Whitney U test, or Chi-square test, as appropriate. Univariate logistic regression analyses were performed to test associations between demographics, acute COVID-19 characteristics and comorbidities with the presence of muscle weakness at visit 1. Subsequently, to explore possible independent risk factors for quadriceps muscle weakness at visit 1, we performed a multivariate backward stepwise logistic regression (with a removal criterion of *p* > 0.05) including age, gender and all other variables that were statistically significant (*p* < 0.10) in univariate analyses. Pearson and Spearman’s correlations were performed, as appropriate, between outcomes assessed at visit 1 and muscle strength at visit 1. Changes in quadriceps muscle strength and prevalence of muscle weakness were assessed using linear mixed model and longitudinal logistic regression, respectively. Both methods enable the use of all available measurements of all subjects. Mean quadriceps muscle strength at the 3 visits was estimated for both cohorts separately using the restricted maximum likelihood method and reported as estimated marginal means with 95% confidence interval (CI). In addition, estimated proportions for peripheral muscle weakness at the 3 visits were reported for both cohorts using generalized linear mixed models. If a statistically significant overall effect of visit was observed, pairwise post hoc tests comparing the visits were performed applying Bonferroni correction using an overall alpha of 5%. As this was an explorative study we did not perform a formal power analysis. Analyses were performed using SPSS statistical software program (IBM, New York, NY, USA), version 25.0, and a *p*-value of <0.05 was considered significant.

## 3. Results

### 3.1. Post-Hospitalized Patients

**Figure 1:**
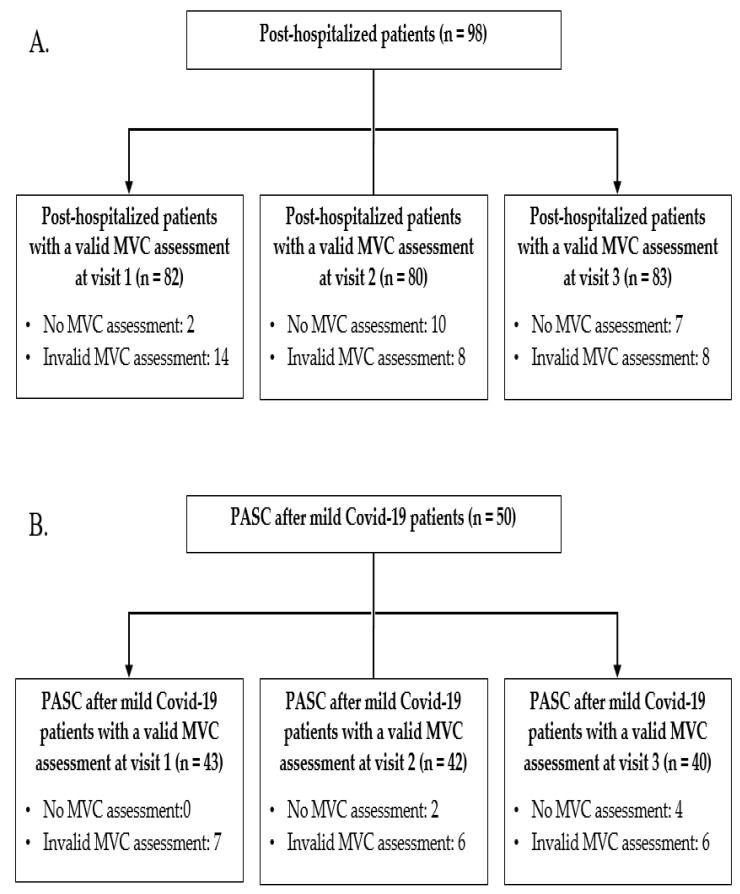
Flowchart of post-hospitalized (**A**) and PASC after mild COVID-19 (**B**) patient inclusion. Abbreviations: MVC: maximal voluntary contraction; PASC: post-acute sequelae of COVID-19.

A total of 98 post-hospitalized patients with proof of previous COVID-19 were considered for inclusion. Of these, valid quadriceps strength assessments were available for 82 patients at visit 1 (Figure 1A). At visit 1, which took place at a median of 10 (8–11) weeks following discharge from the hospital, the mean MVC was 63 ± 18% of predicted and muscle weakness was observed in 59%. Demographics, acute COVID-19 characteristics, and comorbidities of patients with and without muscle weakness at visit 1 are depicted in Table 1. Compared to patients with normal MVC, those with muscle weakness had a significantly lower prevalence of asthma (*p* < 0.05) and a higher prevalence of diabetes mellitus (*p* < 0.01). In univariate logistic analyses, muscle weakness was associated with a lower prevalence of asthma (OR 0.22, 95%CI 0.05–0.89, *p* = 0.03), higher prevalence of diabetes mellitus (OR 15.0, 95%CI 1.87–120.2, *p* = 0.01), and showed trends towards associations with a higher number of comorbidities (OR 1.43, 95%CI 0.96–2.11, *p* = 0.08) and longer hospital length of stay (OR 1.03, 95%CI 1.00–1.06, *p* = 0.05). Subsequently, age, gender, hospital length of stay, comorbid asthma and diabetes mellitus, and a number of comorbidities were entered in a multivariate logistic backward regression analysis (Table 2). Of these, diabetes mellitus and hospital length of stay were retained in the final model as possible risk factors for muscle weakness at visit 1. Furthermore, at visit 1, patients with muscle weakness had significantly lower 6-min walking test distance (6MWD), lower SF-36 physical functioning scores, and lower vital capacity (all *p* < 0.05) (Table 3), and, correspondingly, MVC as percentage of the predicted value correlated significantly with 6MWD (r = 0.54, *p* < 0.01), SF-36 physical functioning scores (ρ = 0.33, *p* < 0.01), and vital capacity (r = 0.28, *p* = 0.01).

**Figure 2:**
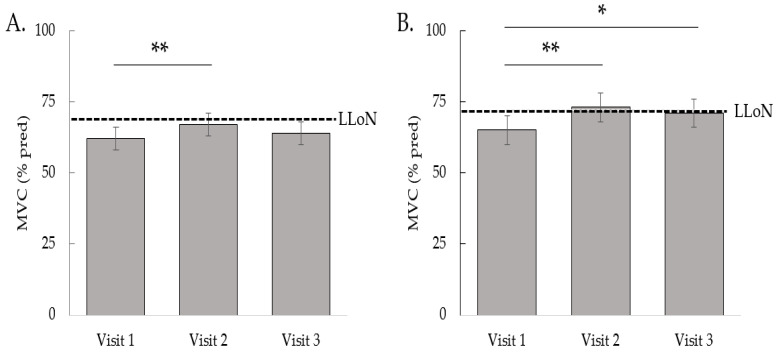
Bar graphs displaying the estimated marginal means of the maximal voluntary contraction (MVC) expressed as percentage of the predicted value at the three visits in post-hospitalized patients (**A**) and in patients with PASC after mild COVID-19 (**B**). Error bars represent 95% CI and dashed lines represent cohort-specific mean lower limit of normal (LLoN). * *p* < 0.05, ** *p* < 0.01.

In addition to visit 1 at 10 (8–11) weeks after hospital discharge, muscle strength was assessed in post-hospitalized patients at 27 (25–29) and 60 (56–64) weeks after hospital discharge (visit 2 and 3, respectively). MVC as a percentage of the predicted value increased by 5% (95%CI 2–8%) between visits 1 and 2 (*p* < 0.01) and remained unchanged between visits 2 and 3 (Figure 2A). Over the three consecutive visits, the estimated proportions of muscle weakness were 0.60, 0.55, and 0.56, respectively (*p* = 0.78).

### 3.2. Patients with PASC after Mild COVID-19

A total of 50 patients with PASC after mild COVID-19 with proof of previous COVID-19 were considered for inclusion. Of these, valid quadriceps muscle strength assessments were available for 43 patients at visit 1 (Figure 1B). At visit 1, which took place at a median of 20 (16–25) weeks after onset of COVID-19 symptoms, the mean MVC was 66 ± 14% of predicted and muscle weakness was observed in 65%. Demographics and comorbidities of patients with and without muscle weakness at visit 1 are depicted in Table 1 and revealed no statistical differences. There were no significant univariate logistic associations between demographics and comorbidities with the presence of muscle weakness at visit 1. At visit 1, patients with muscle weakness had significantly lower 6MWD and higher CIS-fatigue scores (both *p* < 0.05) (Table 3), and, correspondingly, MVC correlated significantly with 6MWD (r = −0.51, *p* < 0.01) and CIS-fatigue score (ρ = −0.40, *p* < 0.01).

In addition to visit 1 at 21 (16–25) weeks after symptom onset of acute COVID-19, muscle strength was evaluated in patients with PASC after mild COVID-19 at 41 (36–49) and 79 (69–85) weeks after symptom onset of acute COVID-19 (visit 2 and 3, respectively). MVC as percentage of the predicted value increased by 8% (95%CI 3–13%) (*p* < 0.01) between visit 1 and 2, and by 6% (95%CI 1–10) (*p* = 0.01) between visit 1 and 3 (Figure 2B). Over the three consecutive visits, the estimated proportions of muscle weakness were 0.67, 0.52, and 0.54, respectively (*p* = 0.32).

### 3.3. Muscle Ultrasound

Muscle ultrasound was performed in 14 post-COVID-19 patients (5 post-hospitalized and 9 PASC after mild COVID-19) with quadriceps muscle weakness. These patients had a mean age of 47 ± 15 years, 64% were male, mean BMI of 26 ± 3 kg/m^2^, and mean MVC of 124 ± 34 Nm (53 ± 9% of predicted). Muscle ultrasound was performed at a median of 43 (33–61) weeks after onset of COVID-19 symptoms, and a median of 13 (8–17) weeks after muscle strength assessment. Muscle thickness and echo intensity were within the limits of normal for all of the investigated muscles in the upper and lower extremities. Average z-scores for the different muscles ranged between −1.0 to 0.9 for muscle thickness, and between −1.3 to 0.5 for echo intensity (Table 4). One patient had an increased echo intensity of the m. vastus lateralis, in whom no neuromuscular abnormalities were found upon more extensive evaluation.

## 4. Discussion

This study reports a high prevalence of quadriceps muscle weakness both in patients discharged from COVID-19 hospitalization and in patients with PASC following mild COVID-19. Despite a small increase in MVC during the ~1.5 years follow-up, the prevalence of muscle weakness remained unchanged in both cohorts. The clinical relevance of muscle weakness was exemplified by significant correlations of MVC with 6MWD, subjective physical complaints and fatigue. Furthermore, diabetes mellitus and hospital length of stay were identified as possible risk factors for quadriceps muscle weakness ~10 weeks after discharge from COVID-19 hospitalization, whereas in those with PASC after mild COVID-19 we could not identify any predictors of muscle weakness from the available data. In addition, in a subgroup of post-COVID-19 patients with objectified quadriceps muscle weakness, muscle ultrasound revealed no large structural abnormalities.

The strengths of our study were the longitudinal and objective assessment of quadriceps muscle strength in two cohorts of post-COVID-19 patients with different severity grades of COVID-19. An important limitation of our study is the lack of muscle strength data *before* (or at the time of) COVID-19 diagnosis or the lack of control groups. Therefore, we cannot exclude the possibility that muscle weakness was pre-existent and that these weaker individuals were more likely to be hospitalized, for which substantial evidence exists when it concerns all-cause hospitalizations [27] and also seems to contribute to COVID-19 hospitalization risk [28]. Whether such reasoning could also apply to patients with PASC following mild COVID-19 remains unknown. Another inherent limitation of our methodology was that differences in sampling and in the timing of the visits precluded direct comparisons between the two cohorts. Indeed, whereas the post-hospitalization cohort was regularly invited after discharge for control visits at the aftercare facility, those with PASC following mild COVID-19 were referred by their general practitioners and thus comprised a selected and heterogenous subgroup that is not representative of all patients who had mild COVID-19.

Muscle weakness following COVID-19 has been proposed to be multifactorial in aetiology in which systemic inflammation, muscle disuse, hypoxaemia, malnutrition, adverse effects of medication, as well as possible SARS-CoV-2 muscular infiltration via the angiotensin-converting enzyme-2 expressed in human skeletal muscle [29], have been proposed [6]. Likely, such factors differentially contribute to muscle weakness across COVID-19 severity grades and depend on hospitalization status and relate to comorbidities. Generally, muscle weakness is common in patients with multimorbidity [30] and diabetes mellitus [31], and contributes to hospitalization risk [27]. Therefore, it is possible that the high prevalence of muscle weakness, and the identification of diabetes mellitus as possible risk factors for muscle weakness, at least partly reflect pre-existent muscle weakness rather than completely invoked by an interaction between SARS-CoV-2 infection and comorbidities. To provide more clarity, future studies are required in which pre-COVID-19 muscle function data are available. Still, considering that length of stay significantly contributed to the risk of muscle weakness, albeit with a small effect (OR = 1.03 per day of hospitalisation), this does suggest an extra insult on muscle strength in those hospitalized for COVID-19. This adds to the many arguments to prevent hospitalization and shorten the length of stay whenever possible. Clearly, those with PASC following mild COVID-19 had fewer comorbidities, and, otherwise, we had no reason to suspect pre-existent muscle weakness in this cohort, although pre-COVID-19 measurements were lacking. It can be hypothesized that even within this subgroup of patients after mild, non-hospitalized, COVID-19, there is variation in severity or characteristics of acute infection relevant to muscle strength, but we did not have the data to support this.

The overall poor improvement of muscle strength was evident. Notably, the high prevalence of muscle weakness did not coincide with low FFMi in either cohort, or with decreased muscle thickness or increased muscle echo intensity in a subgroup with muscle weakness. These findings suggest that muscle atrophy and large structural muscle abnormalities resulting from fibrosis, inflammation, or fat infiltration are not likely to represent the main explanations for post-COVID-19 muscle weakness. Although bioelectrical impedance analysis could have modestly overestimated lean mass [32], we do not believe that the degree of this possible overestimation could have a masked loss of muscle mass that would account for the high prevalence of muscle weakness. Due to logistical constraints inflicted by the disruptive COVID-19 pandemic, there was an average time interval of three months between quadriceps muscle strength measurement and ultrasound assessment. We cannot completely rule out improvement in muscle strength in the meantime. Muscle ultrasound is a reliable technique to detect large structural muscle abnormalities, as frequently demonstrated in neuromuscular diseases [33,34,35,36,37]. However, non-structural changes related to metabolic or mitochondrial alterations, impaired muscle fibre excitation, contractile dysfunction, or deconditioning may go undetected using muscle ultrasound and can therefore not be excluded in our study population. To this end, a pioneer study in post-COVID-19 patients up to 14 months after acute infection has recently shown a variety of histopathological changes in skeletal muscle biopsies including basal lamina duplication, fibre damage, mitochondrial changes, inflammation, and capillary injury [38]. Clearly, considering the scale and the apparent chronicity of the matter, there is a need to increase our knowledge on the pathophysiology of muscle weakness after the full spectrum of COVID-19 severity grades, which should provide insights required to improve muscle strength recovery strategies.

It should be noted that the patients in our study contracted COVID-19 early in the pandemic—i.e., before SARS-CoV-2 variants of concern became dominant and before vaccines became available–, and that during subsequent COVID-19 waves several factors probably relevant to skeletal muscle function have changed. For example, as compared to first-wave patients, those in later waves had better access to both in-hospital and outpatient allied health care and rehabilitation therapy. Furthermore, as the pandemic continues, physical training strategies have been improving [39] which, ideally, would suggest improved recovery trajectories in subsequent cohorts. A potentially less favourable development from the perspective of skeletal muscle, however, is the later introduction of corticosteroids to the treatment for oxygen-dependent hospitalized patients with COVID-19 [40]. Indeed, although corticosteroids have been shown to improve short-term survival and modestly reduce length of stay [40], the well-known deleterious effects of corticosteroids on skeletal muscle [41] should not be overlooked. For these reasons, our findings may not be representative to COVID-19 patients from later waves.

In conclusion, we report high prevalence of clinically relevant muscle weakness with poor long-term improvement in patients previously hospitalized for COVID-19 and in those with PASC following mild COVID-19. Hospital length of stay and diabetes mellitus were identified as possible risk factors for muscle weakness in post-hospitalized patients. In addition, muscle ultrasound could not detect large structural abnormalities in a subgroup of post-COVID-19 patients with quadriceps muscle weakness. Future studies with relevant control groups are warranted to confirm our data.

## Figures and Tables

**Table 1:** Demographics, acute COVID-19 characteristics and comorbidities of post-hospitalized and PASC after mild COVID-19 patients stratified by muscle weakness.

		Post-Hospitalized (n = 82)		PASC after Mild COVID-19 (n = 43)	
	Patients with Missing Data, n	Normal Muscle Strength (n = 34)	Muscle Weakness (n = 48)	Normal Muscle Strength (n = 15)	Muscle Weakness (n = 28)
**Demographics**					
Age, years	0	61 ± 10	60 ± 10	53 ± 17	50 ± 15
Male gender, No. (%)	0	21 (62)	34 (71)	7 (47)	9 (32)
Active smokers, No. (%)	6	0 (0)	0 (0)	0 (0)	0 (0)
**Acute COVID-19 characteristics**					
Length of stay, days	0	10 (4–19)	13 (6–33)	NA	NA
ICU admission, No. (%)	0	11 (32)	19 (40)	NA	NA
CT severity score	15	13 ± 5	14 ± 5	NA	NA
Peak D-Dimer, µg/L	13	1420 (1025–5025)	3580 (810–12,005)	NA	NA
Peak CRP, mg/L	7	115 (50–174)	167 (67–254)	NA	NA
Peak Ferritin, µg/L	10	1184 (618–2550)	1929 (820–3526)	NA	NA
Chloroquine, No. (%)	0	26 (77)	35 (73)	NA	NA
Corticosteroids, No. (%)	0	2 (6)	7 (15)	NA	NA
Anakinra, No. (%)	0	3 (9)	8 (17)	NA	NA
**Comorbidities, No. (%)**					
Cardiovascular	0	8 (24)	11 (23)	1 (7)	5 (18)
Oncological	0	6 (18)	9 (19)	0 (0)	1 (4)
Immunocompromised	0	4 (12)	11 (23)	1 (7)	0 (0)
Chronic lung disease	0	12 (35)	11 (23)	3 (20)	4 (14)
COPD	0	4 (12)	2 (4)	1 (7)	0 (0)
Asthma	0	8 (24)	3 (6) *	2 (13)	4 (14)
Other lung disease	0	4 (12)	7 (15)	0 (0)	0 (0)
Hypertension	0	9 (27)	18 (38)	3 (20)	5 (18)
Diabetes mellitus	0	1 (3)	15 (31) **	0 (0)	3 (11)
Chronic kidney failure	0	0 (0)	5 (10)	0 (0)	0 (0)
Number of comorbidities, No.	0	1 (0–2)	1 (1–3)	0 (0-1)	0 (0–1)

Abbreviations: PASC: post-acute sequelae of COVID-19; ICU: intensive care unit; CT: computed tomography; CRP: C-reactive protein; COPD: chronic obstructive pulmonary disease; NA: not applicable. Significance levels in post-hospitalized patients: * *p* < 0.05, ** *p* < 0.01.

**Table 2:** Outcomes of logistic backward stepwise regression identifying possible predictors of muscle weakness in post-hospitalized COVID-19 patients.

	Full Model					Final Model				
Variables in the Equation	β	SE	OR	95% CI	*p*	β	SE	OR	95% CI	*p*
Age (years)	−0.05	0.03	0.95	0.90–1.01	0.10					
Gender	0.11	0.54	1.12	0.39–3.21	0.84					
Length of stay (days)	0.03	0.02	1.03	1.00–1.06	0.07	0.03	0.02	1.03	1.00–1.06	0.05
Asthma	−1.57	0.85	0.21	0.04–1.11	0.07					
Diabetes mellitus	2.19	1.12	8.95	1.00–80.16	0.05	2.75	1.07	15.62	1.92–127.08	0.01
Number of comorbidities	0.32	0.24	1.38	0.86–2.23	0.18					
Nagelkerke pseudo R^2^	0.33					0.26				

**Table 3:** Timing of visit 1 relative to acute COVID-19 and outcomes assessed at visit 1 of post-hospitalized and PASC after mild COVID-19 patients stratified by muscle weakness.

		Post-Hospitalized (n = 82)		PASC after Mild COVID-19 (n = 43)	
	Patients with Missing Data, n	Normal Muscle Strength (n = 34)	Muscle Weakness (n = 48)	Normal Muscle Strength (n = 15)	Muscle Weakness (n = 28)
Time since symptom onset, weeks	1	12 (11–13)	13 (11–18)	22 (19–25)	18 (15–24)
Time since discharge, weeks	0	10 (8–10)	10 (8–12)	NA	NA
BMI, kg/m^2^	0	27.2 ± 4.3	28.8 ± 3.7	26.4 ± 4.6	28.9 ± 5.2
FFMi, kg/m^2^	2	19.3 ± 2.9	19.9 ± 2.6	18.0 ± 2.1	18.9 ± 2.7
Abnormal FFMi, No. (%)	2	3 (9)	10 (22)	3 (20)	1 (4)
PS-SGA SF score	3	1 (1–2)	1 (1–3)	2 (1–3)	3 (1–6)
mMRC score	1	1 (0–1)	1 (0–2)	1 (1–2)	1 (1–2)
CIS—fatigue score	1	36 (26–43)	41 (32–51)	44 (38–48)	51 (46–55) ^##^
SF-36 physical functioning score	5	70 (53–85)	58 (31–74) *	70 (55–80)	60 (41–75)
FEV_1_, % of predicted	0	99 ± 20	93 ± 18	90 ± 18	100 ± 13
VC, % of predicted	0	103 ± 18	92 ± 19 *	98 ± 9	100 ± 11
DLco, % of predicted	3	80 ± 19	72 ± 18	89 ± 18	91 ± 12
6MWD, m	7	549 ± 119	454 ± 111 **	575 ± 84	516 ± 64 ^#^
6MWD, % of predicted	7	100 ± 19	81 ± 17 **	103 ± 20	89 ± 11 ^#^
CRP > 5 mg/L, No. (%)	1	4 (12)	8 (17)	1 (7)	5 (18)

Abbreviations: PASC: post-acute sequelae of COVID-19; BMI: body mass index; FFMi: fat-free mass index; PS-SGA SF: patient-generated subjective global assessment short form; mMRC: modified Medical Research Council; CIS: Checklist Individual Strength; SF-36: 36-item Short Form Health Survey; FEV_1_: forced expiratory volume in 1 s; VC: vital capacity; DLco: diffusion capacity for carbon monoxide; 6MWD: 6-min walk test distance; CRP: C-reactive protein; NA: not applicable. Significance levels in post-hospitalized patients: * *p* < 0.05, ** *p* < 0.01. Significance levels in PASC after mild COVID-19 patients: ^#^ *p* < 0.05, ^##^ *p* < 0.01.

**Table 4:** Muscle ultrasound data of fourteen post-COVID-19 patients with peripheral muscle weakness.

	Muscle Thickness			Echo Intensity		
	Thickness (cm)	z-Score	Abnormal (n)	Intensity (Grey Level)	z-Score	Abnormal (n)
m. biceps brachii	2.6 (2.2, 3.2)	0.5 (−0.3, 1.7)	0	61 (54,69)	−1.1 (−1.5, −0.5)	0
m. deltoideus	2.0 (1.8, 2.2)	0.3 (−0.3, 0.6)	0	59 (55, 69)	0.3 (−0.4, 0.8)	0
m. flexor carpi radialis	1.3 (1.2, 1.6)	0.3 (−0.4, 1.6)	0	50 (45, 54)	−0.4 (−0.8, 0.1)	0
m. gastrocnemius (medial head)	1.8 (1.6, 1.9)	0.8 (0.1, 1.0)	0	64 (55, 70)	−0.7 (−1.3, −0.1)	0
m. interosseus dorsalis I	1.2 (1.0, 1.3)	0.2 (−0.3, 0.9)	0	44 (39, 48)	−1.3 (−1.7, −0.6)	0
m. peroneus tertius	1.6 (1.5, 1.9)	0.9 (0.6, 2.1)	0	74 (68, 77)	−0.1 (−0.7, 0.2)	0
m. rectus femoris *	4.1 (3.6, 4.7)	−0.2 (−1.4, 0.1)	0	63 (51, 69)	−0.2 (−1.3, 0.6)	0
m. tibialis anterior	2.6 (2.3, 2.9)	0.1 (−0.8, 0.6)	0	82 (74, 85)	0.5 (−0.4, 1.0)	0
m. vastus lateralis	3.5 (2.8, 3.8)	−1.0 (−1.3, −0.2)	0	70 (63, 75)	0.0 (−0.2, 1.1)	1

Data are presented as median (IQR) unless stated otherwise. * 5 patients with missing data.

## Data Availability

The data presented in this study are available on request from the corresponding author. The data are not publicly available due to ethical restrictions.
